# Crystal structures of (*E*)-(3-ethyl-1-methyl-2,6-di­phenyl­piperidin-4-yl­idene)amino phenyl carbonate and (*E*)-(3-isopropyl-1-methyl-2,6-di­phenyl­piperidin-4-yl­idene)amino phenyl carbonate

**DOI:** 10.1107/S1600536814018893

**Published:** 2014-09-13

**Authors:** B. Raghuvarman, R. Sivakumar, V. Thanikachalam, S. Aravindhan

**Affiliations:** a Department of Physics, Presidency College (Autonomous), Chennai 600 005, India; b Department of Chemistry, Annamalai University, Annamalai Nagar, Chidambaram 608 002, India

**Keywords:** crystal structure, piperidine, oxime, 2,6-di­phenyl­piperidine

## Abstract

In the title compounds, C_27_H_28_N_2_O_3_, (I), and C_28_H_30_N_2_O_3_, (II), the conformation about the C=N bond is *E*. The piperidine rings adopt chair conformations with the attached phenyl rings almost normal to their mean planes, the dihedral angles being 85.82 (8) and 85.84 (7)° in (I), and 87.98 (12) and 86.42 (13)° in (II). The phenyl rings are inclined to one another by 52.87 (8)° in (I) and by 60.51 (14)° in (II). The main difference in the conformation of the two compounds is the angle of inclination of the phen­oxy­carbonyl ring to the piperidine ring mean plane. In (I), these two planes are almost coplanar, with a dihedral angle of 2.05 (8)°, while in (II), this angle is 45.24 (13)°. In the crystal of (I), mol­ecules are linked by C—H⋯O hydrogen bonds, forming inversion dimers with *R*_2_^2^(14) loops. The dimers are linked *via* C—H⋯π inter­actions forming a three-dimensional network. In the crystal of (II), there are no significant inter­molecular inter­actions present.

## Abstract 2

The title compounds are the 3-ethyl, (I), and 3-isopropyl, (II), derivatives of (1-methyl-2,6-diphenylpiperidin-4-ylidene)amino phenyl carbonate. The main difference in the conformation of the two compounds is the angle of inclination of the phenoxycarbonyl ring with respect to the piperidine ring mean plane, with a dihedral angle of 2.05 (8)° in (I) and 45.24 (13)° in (II).

## Chemical context   

Piperidine derivatives are one of the simplest heterocyclic units found in nature, for example in several alkaloids. Such compounds have been used as anti­histamines, anaesthetics, tranquilizers and hypotensive agents (Robinson, 1973 ▶). The synthesis and biological activity of piperidin-4-one derivatives has received considerable attention (Parthiban *et al.*, 2009 ▶; Narayanan *et al.*, 2012 ▶). Both natural and synthetic piperidine derivatives have high pharmaceutical value, hence our inter­est in the synthesis of 2,6-disubstituted piperidine derivatives. We report herein on the synthesis and crystal structures of (*E*)-(3-ethyl-1-methyl-2,6-di­phenyl­piperidin-4-yl­idene)amino phenyl carbonate, (I), and (*E*)-(3-isopropyl-1-methyl-2,6-di­phenyl­piperidin-4-yl­idene)amino phenyl carbonate, (II).[Chem scheme1] 
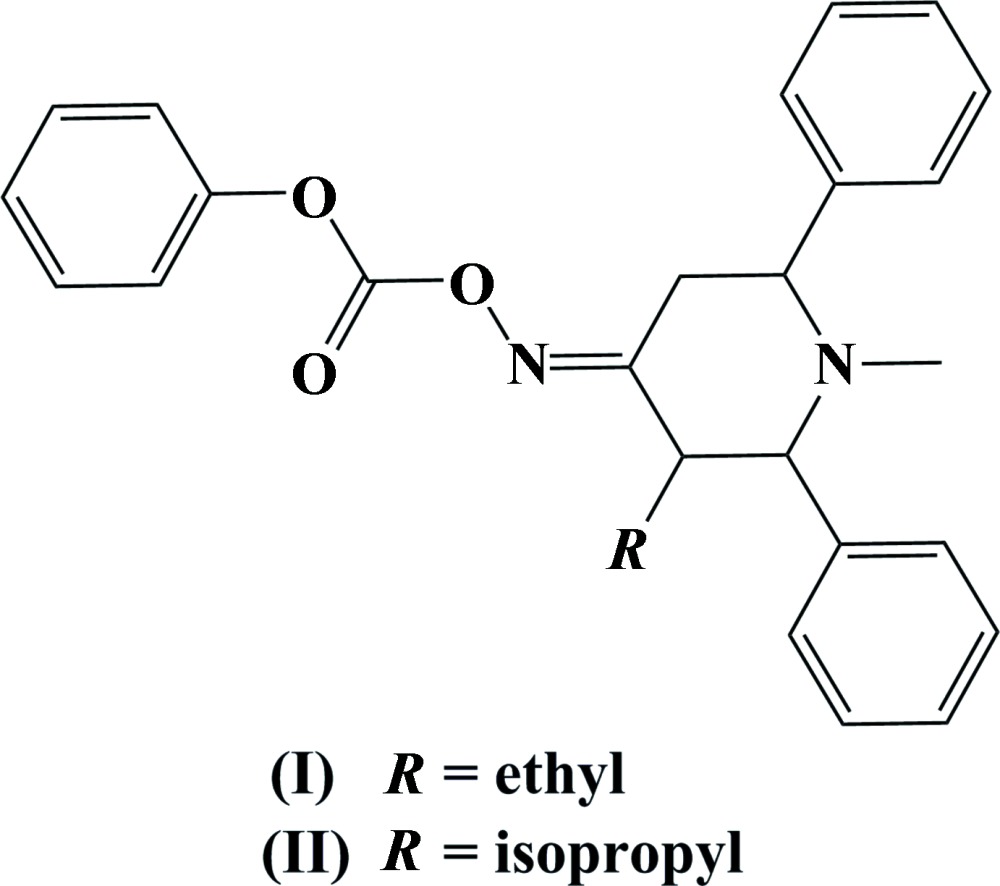


## Structural commentary   

**Figure 1:**
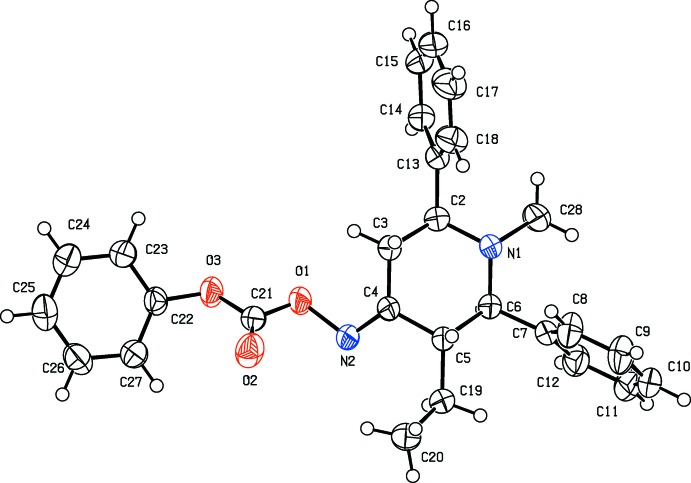
The mol­ecular structure of compound (I), with the atom labelling. Displacement ellipsoids are drawn at the 50% probability level.

The mol­ecular structure of compound (I) is shown in Fig. 1 ▶. The piperidine ring adopts a chair conformation. The attached phenyl rings (C7–C12 and C13–C18) are twisted away from the mean plane of the piperidine ring by 85.82 (8) and 85.84 (7)°. The two phenyl rings are oriented to each other with a dihedral angle of 52.87 (8)°. The phen­oxy ring (C22–C27) is almost coplanar with the piperidine ring mean plane with a dihedral angle of 2.05 (8)°. The sum of the bond angles around atom N1 (331.9°) is in accordance with *sp* ^3^ hybridization. The ethyl group substituted at position 5 of the piperidine moiety is in an equatorial orientation.

**Figure 2:**
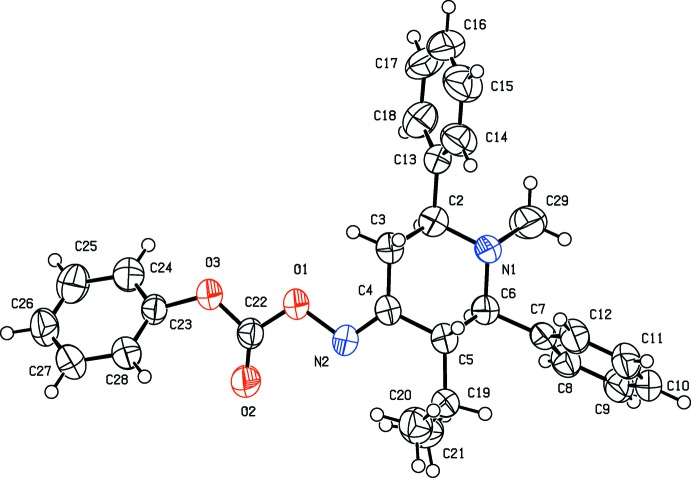
The mol­ecular structure of compound (II), with the atom labelling. Displacement ellipsoids are drawn at the 50% probability level.

The mol­ecular structure of compound (II) is shown in Fig. 2 ▶. The piperidine ring also adopts a chair conformation. The attached phenyl rings (C7—C12 and C13—C18) are twisted away from the mean plane of the piperidine ring by 87.98 (12) and 86.42 (13) °. The two phenyl rings are oriented to each other with a dihedral angle of 60.51 (14)°. In (II) the phen­oxy ring (C23–C28) is no longer coplanar with the mean plane of the piperidine ring but inclined to it by 45.24 (13)°. The sum of the bond angles around atom N1 (335.6°) is in accordance with *sp* ^3^ hybridization. The isopropyl group substituted at position 5 of the piperidine moiety is in an equatorial orientation.

For both compounds (I) and (II), the bond lengths and bond angles are comparable with the values reported for the 3-methyl derivative (III), (*E*)-3-methyl-1-methyl-2,6-di­phenyl­piperidin-4-one *O*-phen­oxy­carbonyl oxime (Raghuvarman *et al.*, 2014 ▶). The overall conformation of compound (III) is very similar to that of compound (II), with the phen­oxy ring inclined to the mean plane of the piperidine ring by 32.79 (9)°, compared to 45.24 (13)° in (II).

## Supra­molecular features   

**Figure 3:**
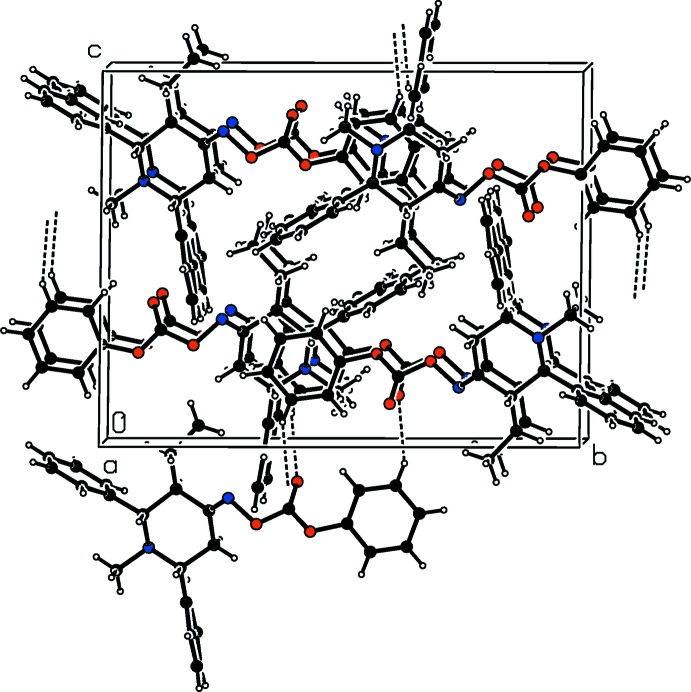
A view along the *a* axis of the crystal packing of compound (I). The C—H⋯O hydrogen bonds are shown as dashed lines (see Table 1 ▶ for details).

In the crystal of (I), pairs of C—H⋯O hydrogen bonds link the mol­ecules, forming inversion dimers with 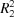
(14) loops. The dimers are linked *via* C-H⋯π inter­actions, forming a three-dimensional network (Fig. 3 ▶ and Table 1 ▶).

**Figure 4:**
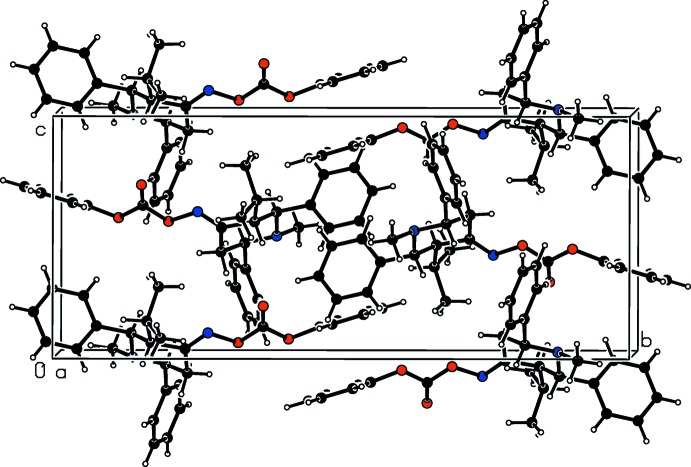
A view along the *a* axis of the crystal packing of compound (II).

In the crystal of (II), there are no significant inter­molecular inter­actions present. This is similar to the situation in the crystal of compound (III). The packing in (II) is illustrated in Fig. 4 ▶.

## Database survey   

A search of the Cambridge Structural Database (Version 5.35, last update May 2014; Allen, 2002 ▶) revealed the presence of 25 structures with the substructure 2,6-diphenyl-4-piperidine oxime. Of these, 16 have the piperidine ring in a chair conformation, while seven have a boat conformation and two a screw-boat conformation. In the various structures, the diphenyl rings are inclined to one another by dihedral angles varying from *ca*. 44.9° in a very similar compound to those studied here, *viz* (*E*)-{[(3-isopropyl-1-methyl-2,6-di­phenylpip­eridin-4-yl­idene)amino]­oxy}(pyridin-3-yl)methanone (CCDC refcode: HOFFIT; Vinuchakkaravarthy *et al.*, 2014 ▶), to *ca*. 80.7° in *t*-3-benzyl-*r*-2,*c*-6-bis­(4-meth­oxy­phen­yl)piper­idin-4-one oxime (CCDC refcode: HODGAU; Jayabharathi *et al.*, 2008 ▶).

## Synthesis and crystallization   

Compounds (I) and (II) were synthesized by Mannich condensation using benzaldehyde (2 mol), ammonium acetate (1 mol) and methyl propyl ketone (1 mol) for (I), and methyl isobutyl ketone (1 mol) for (II), in absolute ethanol. The mixtures were warmed for 30 min and stirred overnight at room temperature. The products obtained were treated with methyl iodide (1.5 mol) in the presence of potassium carbon­ate (2 mol) in acetone (10 ml) and refluxed to give 1-methyl-3-ethyl-2,6-di­phenyl­piperidin-4-one and 1-methyl-3-isopropyl-2,6-di­phenyl­piperidin-4-one, respectively. The oximations were carried out using hydroxyl­amine hydro­chloride (2 mol) in the presence of sodium acetate (2 mol) in ethanol (10 ml) and refluxed. To the resulting oximes, (0.5 g, 1.62 mmol) for the precursor of (I) and (0.5 g, 1.55 mmol) for the precursor of (II), in dry tetra­hydro­furan (10 ml), was added potassium carbonate (0.48 g, 3.24 mmol) followed by tetra­butyl­ammonium bromide (0.58 g, 1.62 mmol). After stirring for 15 min, phenyl chloro­formate (0.38 g, 2.43 mmol) was added dropwise to the reaction mixtures over a period of 15 min. The mixtures were stirred at ambient temperature for 2 h and progress of the reactions was monitored by thin-layer chromatography. Upon completion of the reactions, the reaction mixtures were diluted with water (20 ml) and extracted with di­chloro­methane (2 × 20 ml). The combined organic layers were washed with water (2 × 20 ml), brine solution (20 ml), dried over anhydrous sodium sulfate (5 g), filtered and concentrated under reduced pressure. The crude products were purified by column chromatography over silica gel (100–200 mesh) eluted with a solvent system of ethyl acetate–petroleum ether (2:98). The pure fractions were collected and concentrated under reduced pressure to give white solids of (I) (yield 0.60 g, 86%) and (II) (yield 0.56 g, 82%), which were recrystallized from a DMF–water mixture (9:1) to give colourless block-like crystals of (I) and (II), respectively.

## Refinement   

Crystal data, data collection and structure refinement details are summarized in Table 2 ▶. The C-bound H atoms were positioned geometrically and allowed to ride on their parent atoms: C–H = 0.93–0.98 Å with *U*_iso_(H) = 1.5*U*_eq_(C-meth­yl) and = 1.2*U*_eq_(C) for other H atoms.

## Supplementary Material

Crystal structure: contains datablock(s) I, II. DOI: 10.1107/S1600536814018893/su2763sup1.cif

Structure factors: contains datablock(s) I. DOI: 10.1107/S1600536814018893/su2763Isup2.hkl

Structure factors: contains datablock(s) II. DOI: 10.1107/S1600536814018893/su2763IIsup3.hkl

Supporting information file. DOI: 10.1107/S1600536814018893/su2763Isup4.cml

Supporting information file. DOI: 10.1107/S1600536814018893/su2763IIsup5.cml

CCDC references: 1020223, 1020224

Additional supporting information:  crystallographic information; 3D view; checkCIF report

## Figures and Tables

**Table 1:** Hydrogen-bond geometry (Å, °) for (I) *Cg*3 and *Cg*4 are the centroids of the C13–C18 and C22–C27 rings, respectively.

*D*—H⋯*A*	*D*—H	H⋯*A*	*D*⋯*A*	*D*—H⋯*A*
C26—H26⋯O2^i^	0.93	2.57	3.422 (2)	153
C6—H6⋯*Cg*4^ii^	0.98	2.99	3.959 (2)	170
C10—H10⋯*Cg*3^iii^	0.93	2.96	3.824 (2)	155

Symmetry codes: (i) 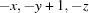; (ii) 
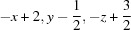
; (iii) 
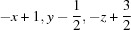
.

**Table 2:** Experimental details

	(I)	(II)
Crystal data		
Chemical formula	C_27_H_28_N_2_O_3_	C_28_H_30_N_2_O_3_
*M*_r_	428.51	442.54
Crystal system, space group	Monoclinic, *P*2_1_/*c*	Monoclinic, *P*2_1_/*c*
Temperature (K)	293	293
*a*, *b*, *c* (Å)	9.3844 (5), 17.8121 (8), 14.4077 (7)	10.3511 (5), 23.9398 (10), 10.0587 (4)
β (°)	107.216 (2)	94.997 (2)
*V* (Å^3^)	2300.4 (2)	2483.11 (19)
*Z*	4	4
Radiation type	Mo *K*α	Mo *K*α
μ (mm^−1^)	0.08	0.08
Crystal size (mm)	0.26 × 0.23 × 0.19	0.28 × 0.25 × 0.20

Data collection		
Diffractometer	Bruker SMART APEXII CCD	Bruker SMART APEXII CCD
Absorption correction	Multi-scan (*SADABS*; Bruker, 2008 ▶)	Multi-scan (*SADABS*; Bruker, 2008 ▶)
*T*_min_, *T*_max_	0.979, 0.985	0.979, 0.985
No. of measured, independent and observed [*I* > 2σ(*I*)] reflections	27700, 6225, 3960	21076, 4150, 2894
*R*_int_	0.038	0.036
(sin θ/λ)_max_ (Å^−1^)	0.687	0.586

Refinement		
*R*[*F* ^2^ > 2σ(*F* ^2^)], *wR*(*F* ^2^), *S*	0.047, 0.132, 1.04	0.052, 0.145, 1.01
No. of reflections	6225	4150
No. of parameters	291	301
H-atom treatment	H-atom parameters constrained	H-atom parameters constrained
Δρ_max_, Δρ_min_ (e Å^−3^)	0.18, −0.21	0.39, −0.17

Computer programs: *APEX2* and *SAINT* (Bruker, 2008 ▶), *SHELXS97*, *SHELXL97* and *SHELXL2013* (Sheldrick, 2008 ▶) and *PLATON* (Spek, 2009 ▶).

## supplementary crystallographic information

### Crystal data

**Table:**

C_28_H_30_N_2_O_3_	*F*(000) = 944
*M**_r_* = 442.54	*D*_x_ = 1.184 Mg m^−^^3^
Monoclinic, *P*2_1_/*c*	Mo *K*α radiation, λ = 0.71073 Å
Hall symbol: -P 2ybc	Cell parameters from 2894 reflections
*a* = 10.3511 (5) Å	θ = 2.6–24.6°
*b* = 23.9398 (10) Å	µ = 0.08 mm^−^^1^
*c* = 10.0587 (4) Å	*T* = 293 K
β = 94.997 (2)°	Block, colourless
*V* = 2483.11 (19) Å^3^	0.28 × 0.25 × 0.20 mm
*Z* = 4	

### Data collection

**Table:**

Bruker SMART APEXII CCD diffractometer	4150 independent reflections
Radiation source: fine-focus sealed tube	2894 reflections with *I* > 2σ(*I*)
Graphite monochromator	*R*_int_ = 0.036
ω and φ scans	θ_max_ = 24.6°, θ_min_ = 2.6°
Absorption correction: multi-scan (*SADABS*; Bruker, 2008)	*h* = −12→11
*T*_min_ = 0.979, *T*_max_ = 0.985	*k* = −27→28
21076 measured reflections	*l* = −11→11

### Refinement

**Table:**

Refinement on *F*^2^	Primary atom site location: structure-invariant direct methods
Least-squares matrix: full	Secondary atom site location: difference Fourier map
*R*[*F*^2^ > 2σ(*F*^2^)] = 0.052	Hydrogen site location: inferred from neighbouring sites
*wR*(*F*^2^) = 0.145	H-atom parameters constrained
*S* = 1.01	*w* = 1/[σ^2^(*F*_o_^2^) + (0.0589*P*)^2^ + 1.1855*P*] where *P* = (*F*_o_^2^ + 2*F*_c_^2^)/3
4150 reflections	(Δ/σ)_max_ < 0.001
301 parameters	Δρ_max_ = 0.39 e Å^−^^3^
0 restraints	Δρ_min_ = −0.17 e Å^−^^3^

### Special details

**Table:**

Geometry. All e.s.d.'s (except the e.s.d. in the dihedral angle between two l.s. planes) are estimated using the full covariance matrix. The cell e.s.d.'s are taken into account individually in the estimation of e.s.d.'s in distances, angles and torsion angles; correlations between e.s.d.'s in cell parameters are only used when they are defined by crystal symmetry. An approximate (isotropic) treatment of cell e.s.d.'s is used for estimating e.s.d.'s involving l.s. planes.
Refinement. Refinement of *F*^2^ against ALL reflections. The weighted *R*-factor *wR* and goodness of fit *S* are based on *F*^2^, conventional *R*-factors *R* are based on *F*, with *F* set to zero for negative *F*^2^. The threshold expression of *F*^2^ > σ(*F*^2^) is used only for calculating *R*-factors(gt) *etc*. and is not relevant to the choice of reflections for refinement. *R*-factors based on *F*^2^ are statistically about twice as large as those based on *F*, and *R*-factors based on ALL data will be even larger.

### Fractional atomic coordinates and isotropic or equivalent isotropic displacement parameters (Å^2^)

**Table:**

	*x*	*y*	*z*	*U*_iso_*/*U*_eq_	
C2	0.0430 (2)	0.18137 (10)	−0.0563 (3)	0.0584 (6)	
H2	0.0064	0.1957	0.0236	0.070*	
C3	0.1492 (2)	0.22117 (10)	−0.0893 (3)	0.0608 (7)	
H3A	0.1787	0.2114	−0.1752	0.073*	
H3B	0.1151	0.2589	−0.0953	0.073*	
C4	0.2604 (2)	0.21879 (9)	0.0150 (2)	0.0525 (6)	
C5	0.3125 (2)	0.16055 (10)	0.0412 (2)	0.0536 (6)	
H5	0.3350	0.1467	−0.0455	0.064*	
C6	0.1996 (2)	0.12406 (10)	0.0777 (2)	0.0547 (6)	
H6	0.1684	0.1396	0.1592	0.066*	
C7	0.2425 (2)	0.06462 (9)	0.1082 (2)	0.0487 (6)	
C8	0.2295 (2)	0.04233 (10)	0.2329 (2)	0.0585 (6)	
H8	0.1936	0.0639	0.2972	0.070*	
C9	0.2691 (3)	−0.01168 (12)	0.2631 (3)	0.0719 (8)	
H9	0.2592	−0.0264	0.3471	0.086*	
C10	0.3228 (3)	−0.04338 (11)	0.1694 (3)	0.0739 (8)	
H10	0.3503	−0.0796	0.1897	0.089*	
C11	0.3358 (3)	−0.02159 (11)	0.0459 (3)	0.0708 (8)	
H11	0.3724	−0.0431	−0.0180	0.085*	
C12	0.2955 (3)	0.03161 (11)	0.0155 (3)	0.0628 (7)	
H12	0.3042	0.0457	−0.0695	0.075*	
C13	−0.0643 (2)	0.18131 (9)	−0.1681 (2)	0.0532 (6)	
C14	−0.0462 (3)	0.15994 (12)	−0.2912 (3)	0.0727 (8)	
H14	0.0343	0.1453	−0.3070	0.087*	
C15	−0.1438 (4)	0.15961 (15)	−0.3913 (3)	0.0894 (10)	
H15	−0.1291	0.1448	−0.4742	0.107*	
C16	−0.2622 (4)	0.18061 (14)	−0.3713 (4)	0.0901 (10)	
H16	−0.3284	0.1803	−0.4400	0.108*	
C17	−0.2831 (3)	0.20193 (14)	−0.2516 (4)	0.0878 (10)	
H17	−0.3643	0.2161	−0.2371	0.105*	
C18	−0.1845 (3)	0.20295 (12)	−0.1497 (3)	0.0721 (8)	
H18	−0.1996	0.2185	−0.0677	0.086*	
C19	0.4372 (2)	0.15494 (10)	0.1351 (2)	0.0551 (6)	
H19	0.4554	0.1148	0.1414	0.066*	
C20	0.5495 (3)	0.18046 (12)	0.0689 (3)	0.0746 (8)	
H20A	0.5565	0.1628	−0.0159	0.112*	
H20B	0.6284	0.1750	0.1248	0.112*	
H20C	0.5345	0.2197	0.0559	0.112*	
C21	0.4315 (3)	0.17488 (12)	0.2767 (3)	0.0756 (8)	
H21A	0.4220	0.2148	0.2773	0.113*	
H21B	0.5101	0.1646	0.3287	0.113*	
H21C	0.3589	0.1579	0.3142	0.113*	
C22	0.2968 (2)	0.35449 (9)	0.1078 (2)	0.0500 (6)	
C23	0.2503 (2)	0.45192 (9)	0.1050 (2)	0.0498 (6)	
C24	0.1424 (3)	0.48133 (11)	0.1271 (3)	0.0675 (7)	
H24	0.0610	0.4647	0.1159	0.081*	
C25	0.1551 (3)	0.53614 (13)	0.1665 (3)	0.0823 (9)	
H25	0.0818	0.5569	0.1818	0.099*	
C26	0.2743 (4)	0.56017 (12)	0.1832 (3)	0.0806 (9)	
H26	0.2825	0.5973	0.2096	0.097*	
C27	0.3816 (3)	0.52999 (12)	0.1614 (3)	0.0753 (8)	
H27	0.4631	0.5465	0.1738	0.090*	
C28	0.3704 (3)	0.47520 (11)	0.1212 (3)	0.0627 (7)	
H28	0.4435	0.4545	0.1054	0.075*	
C29	−0.0141 (3)	0.08902 (14)	0.0065 (4)	0.1030 (12)	
H29A	−0.0487	0.1021	0.0863	0.155*	
H29B	0.0178	0.0516	0.0201	0.155*	
H29C	−0.0811	0.0894	−0.0658	0.155*	
N1	0.09137 (17)	0.12532 (8)	−0.02577 (19)	0.0518 (5)	
N2	0.31261 (19)	0.25920 (8)	0.0822 (2)	0.0552 (5)	
O1	0.24663 (16)	0.31067 (6)	0.03989 (17)	0.0584 (4)	
O2	0.3785 (2)	0.35587 (7)	0.19522 (19)	0.0783 (6)	
O3	0.22803 (16)	0.39771 (6)	0.05421 (17)	0.0614 (5)	

### Atomic displacement parameters (Å^2^)

**Table:**

	*U*^11^	*U*^22^	*U*^33^	*U*^12^	*U*^13^	*U*^23^
C2	0.0579 (15)	0.0519 (15)	0.0644 (15)	0.0002 (12)	−0.0010 (12)	0.0028 (12)
C3	0.0659 (16)	0.0453 (14)	0.0689 (16)	−0.0064 (12)	−0.0066 (13)	0.0039 (12)
C4	0.0537 (14)	0.0407 (13)	0.0622 (15)	−0.0064 (11)	−0.0007 (11)	0.0020 (11)
C5	0.0546 (14)	0.0428 (13)	0.0625 (15)	−0.0039 (11)	0.0004 (11)	−0.0012 (11)
C6	0.0556 (14)	0.0465 (13)	0.0607 (14)	−0.0052 (11)	−0.0035 (11)	0.0059 (11)
C7	0.0493 (13)	0.0426 (13)	0.0524 (13)	−0.0082 (10)	−0.0054 (11)	0.0023 (11)
C8	0.0667 (16)	0.0532 (15)	0.0554 (15)	−0.0121 (12)	0.0047 (12)	0.0002 (12)
C9	0.095 (2)	0.0556 (17)	0.0611 (16)	−0.0195 (15)	−0.0130 (15)	0.0153 (14)
C10	0.081 (2)	0.0407 (15)	0.094 (2)	−0.0037 (13)	−0.0266 (17)	0.0028 (16)
C11	0.0756 (19)	0.0509 (17)	0.085 (2)	−0.0036 (14)	0.0024 (15)	−0.0132 (15)
C12	0.0779 (18)	0.0539 (16)	0.0561 (15)	−0.0114 (13)	0.0033 (13)	−0.0013 (13)
C13	0.0513 (14)	0.0430 (13)	0.0640 (15)	0.0014 (11)	−0.0017 (11)	0.0025 (11)
C14	0.0610 (17)	0.0781 (19)	0.0781 (19)	0.0008 (14)	0.0005 (14)	−0.0201 (16)
C15	0.101 (3)	0.093 (2)	0.0714 (19)	−0.011 (2)	−0.0080 (18)	−0.0134 (17)
C16	0.084 (2)	0.086 (2)	0.094 (3)	−0.0046 (19)	−0.031 (2)	0.024 (2)
C17	0.0569 (18)	0.088 (2)	0.116 (3)	0.0213 (16)	−0.0047 (18)	0.024 (2)
C18	0.0718 (19)	0.0697 (18)	0.0758 (18)	0.0124 (15)	0.0129 (15)	0.0058 (15)
C19	0.0481 (13)	0.0451 (14)	0.0704 (16)	−0.0021 (11)	−0.0047 (11)	0.0002 (12)
C20	0.0632 (17)	0.0665 (18)	0.095 (2)	−0.0030 (14)	0.0126 (15)	−0.0041 (16)
C21	0.084 (2)	0.0757 (19)	0.0645 (17)	0.0037 (16)	−0.0079 (14)	−0.0003 (15)
C22	0.0591 (14)	0.0406 (13)	0.0501 (13)	−0.0032 (11)	0.0037 (12)	0.0031 (11)
C23	0.0626 (15)	0.0379 (12)	0.0478 (13)	−0.0020 (11)	−0.0006 (11)	0.0019 (10)
C24	0.0640 (17)	0.0564 (16)	0.0816 (18)	−0.0004 (13)	0.0029 (14)	0.0005 (14)
C25	0.089 (2)	0.0608 (19)	0.097 (2)	0.0181 (17)	0.0090 (18)	−0.0123 (16)
C26	0.108 (3)	0.0470 (16)	0.084 (2)	−0.0017 (17)	−0.0103 (18)	−0.0136 (14)
C27	0.081 (2)	0.0556 (17)	0.086 (2)	−0.0166 (15)	−0.0094 (16)	−0.0041 (15)
C28	0.0621 (16)	0.0511 (15)	0.0742 (17)	−0.0031 (12)	0.0010 (13)	−0.0046 (13)
C29	0.0671 (19)	0.087 (2)	0.149 (3)	−0.0271 (17)	−0.025 (2)	0.050 (2)
N1	0.0469 (11)	0.0426 (11)	0.0643 (12)	−0.0080 (9)	−0.0047 (9)	0.0060 (9)
N2	0.0603 (12)	0.0384 (11)	0.0657 (12)	0.0003 (9)	−0.0020 (10)	0.0038 (10)
O1	0.0662 (11)	0.0382 (9)	0.0683 (10)	−0.0025 (8)	−0.0081 (8)	−0.0025 (8)
O2	0.1028 (15)	0.0504 (11)	0.0750 (12)	−0.0003 (10)	−0.0308 (12)	0.0000 (9)
O3	0.0701 (11)	0.0395 (9)	0.0714 (11)	−0.0018 (8)	−0.0125 (9)	−0.0031 (8)

### Geometric parameters (Å, º)

**Table:**

C2—N1	1.456 (3)	C16—H16	0.9300
C2—C13	1.510 (3)	C17—C18	1.382 (4)
C2—C3	1.513 (3)	C17—H17	0.9300
C2—H2	0.9800	C18—H18	0.9300
C3—C4	1.490 (3)	C19—C21	1.508 (4)
C3—H3A	0.9700	C19—C20	1.517 (4)
C3—H3B	0.9700	C19—H19	0.9800
C4—N2	1.273 (3)	C20—H20A	0.9600
C4—C5	1.510 (3)	C20—H20B	0.9600
C5—C6	1.529 (3)	C20—H20C	0.9600
C5—C19	1.537 (3)	C21—H21A	0.9600
C5—H5	0.9800	C21—H21B	0.9600
C6—N1	1.462 (3)	C21—H21C	0.9600
C6—C7	1.514 (3)	C22—O2	1.166 (3)
C6—H6	0.9800	C22—O1	1.332 (3)
C7—C12	1.373 (3)	C22—O3	1.342 (3)
C7—C8	1.380 (3)	C23—C24	1.355 (3)
C8—C9	1.382 (4)	C23—C28	1.359 (3)
C8—H8	0.9300	C23—O3	1.406 (3)
C9—C10	1.365 (4)	C24—C25	1.374 (4)
C9—H9	0.9300	C24—H24	0.9300
C10—C11	1.365 (4)	C25—C26	1.359 (4)
C10—H10	0.9300	C25—H25	0.9300
C11—C12	1.366 (4)	C26—C27	1.359 (4)
C11—H11	0.9300	C26—H26	0.9300
C12—H12	0.9300	C27—C28	1.375 (4)
C13—C14	1.368 (4)	C27—H27	0.9300
C13—C18	1.375 (4)	C28—H28	0.9300
C14—C15	1.363 (4)	C29—N1	1.454 (3)
C14—H14	0.9300	C29—H29A	0.9600
C15—C16	1.355 (5)	C29—H29B	0.9600
C15—H15	0.9300	C29—H29C	0.9600
C16—C17	1.343 (5)	N2—O1	1.454 (2)
			
N1—C2—C13	111.92 (19)	C16—C17—C18	120.4 (3)
N1—C2—C3	112.6 (2)	C16—C17—H17	119.8
C13—C2—C3	109.8 (2)	C18—C17—H17	119.8
N1—C2—H2	107.4	C13—C18—C17	120.8 (3)
C13—C2—H2	107.4	C13—C18—H18	119.6
C3—C2—H2	107.4	C17—C18—H18	119.6
C4—C3—C2	110.7 (2)	C21—C19—C20	112.4 (2)
C4—C3—H3A	109.5	C21—C19—C5	116.9 (2)
C2—C3—H3A	109.5	C20—C19—C5	109.2 (2)
C4—C3—H3B	109.5	C21—C19—H19	105.8
C2—C3—H3B	109.5	C20—C19—H19	105.8
H3A—C3—H3B	108.1	C5—C19—H19	105.8
N2—C4—C3	127.6 (2)	C19—C20—H20A	109.5
N2—C4—C5	118.7 (2)	C19—C20—H20B	109.5
C3—C4—C5	113.6 (2)	H20A—C20—H20B	109.5
C4—C5—C6	107.51 (19)	C19—C20—H20C	109.5
C4—C5—C19	117.11 (19)	H20A—C20—H20C	109.5
C6—C5—C19	114.9 (2)	H20B—C20—H20C	109.5
C4—C5—H5	105.4	C19—C21—H21A	109.5
C6—C5—H5	105.4	C19—C21—H21B	109.5
C19—C5—H5	105.4	H21A—C21—H21B	109.5
N1—C6—C7	110.88 (19)	C19—C21—H21C	109.5
N1—C6—C5	111.81 (19)	H21A—C21—H21C	109.5
C7—C6—C5	111.7 (2)	H21B—C21—H21C	109.5
N1—C6—H6	107.4	O2—C22—O1	129.3 (2)
C7—C6—H6	107.4	O2—C22—O3	127.3 (2)
C5—C6—H6	107.4	O1—C22—O3	103.40 (19)
C12—C7—C8	118.1 (2)	C24—C23—C28	121.7 (2)
C12—C7—C6	122.0 (2)	C24—C23—O3	115.4 (2)
C8—C7—C6	119.9 (2)	C28—C23—O3	122.8 (2)
C7—C8—C9	120.7 (3)	C23—C24—C25	119.0 (3)
C7—C8—H8	119.6	C23—C24—H24	120.5
C9—C8—H8	119.6	C25—C24—H24	120.5
C10—C9—C8	120.0 (3)	C26—C25—C24	120.2 (3)
C10—C9—H9	120.0	C26—C25—H25	119.9
C8—C9—H9	120.0	C24—C25—H25	119.9
C9—C10—C11	119.6 (3)	C27—C26—C25	120.1 (3)
C9—C10—H10	120.2	C27—C26—H26	120.0
C11—C10—H10	120.2	C25—C26—H26	120.0
C10—C11—C12	120.5 (3)	C26—C27—C28	120.3 (3)
C10—C11—H11	119.7	C26—C27—H27	119.8
C12—C11—H11	119.7	C28—C27—H27	119.8
C11—C12—C7	121.1 (3)	C23—C28—C27	118.7 (3)
C11—C12—H12	119.5	C23—C28—H28	120.7
C7—C12—H12	119.5	C27—C28—H28	120.7
C14—C13—C18	117.4 (2)	N1—C29—H29A	109.5
C14—C13—C2	121.7 (2)	N1—C29—H29B	109.5
C18—C13—C2	121.0 (2)	H29A—C29—H29B	109.5
C15—C14—C13	121.3 (3)	N1—C29—H29C	109.5
C15—C14—H14	119.3	H29A—C29—H29C	109.5
C13—C14—H14	119.3	H29B—C29—H29C	109.5
C16—C15—C14	120.6 (3)	C29—N1—C2	110.3 (2)
C16—C15—H15	119.7	C29—N1—C6	111.9 (2)
C14—C15—H15	119.7	C2—N1—C6	113.42 (18)
C17—C16—C15	119.5 (3)	C4—N2—O1	108.79 (18)
C17—C16—H16	120.3	C22—O1—N2	111.42 (17)
C15—C16—H16	120.3	C22—O3—C23	120.09 (18)
			
N1—C2—C3—C4	−50.5 (3)	C14—C13—C18—C17	1.2 (4)
C13—C2—C3—C4	−175.9 (2)	C2—C13—C18—C17	−178.9 (3)
C2—C3—C4—N2	−125.4 (3)	C16—C17—C18—C13	−1.2 (5)
C2—C3—C4—C5	53.8 (3)	C4—C5—C19—C21	61.7 (3)
N2—C4—C5—C6	123.4 (2)	C6—C5—C19—C21	−65.9 (3)
C3—C4—C5—C6	−55.9 (3)	C4—C5—C19—C20	−67.4 (3)
N2—C4—C5—C19	−7.7 (3)	C6—C5—C19—C20	165.0 (2)
C3—C4—C5—C19	173.0 (2)	C28—C23—C24—C25	0.3 (4)
C4—C5—C6—N1	56.0 (3)	O3—C23—C24—C25	−174.8 (2)
C19—C5—C6—N1	−171.7 (2)	C23—C24—C25—C26	−0.2 (5)
C4—C5—C6—C7	−179.08 (19)	C24—C25—C26—C27	−0.2 (5)
C19—C5—C6—C7	−46.8 (3)	C25—C26—C27—C28	0.6 (5)
N1—C6—C7—C12	65.1 (3)	C24—C23—C28—C27	0.1 (4)
C5—C6—C7—C12	−60.4 (3)	O3—C23—C28—C27	174.9 (2)
N1—C6—C7—C8	−115.4 (2)	C26—C27—C28—C23	−0.6 (4)
C5—C6—C7—C8	119.1 (2)	C13—C2—N1—C29	−56.4 (3)
C12—C7—C8—C9	0.1 (4)	C3—C2—N1—C29	179.4 (2)
C6—C7—C8—C9	−179.4 (2)	C13—C2—N1—C6	177.2 (2)
C7—C8—C9—C10	0.5 (4)	C3—C2—N1—C6	53.0 (3)
C8—C9—C10—C11	−0.6 (4)	C7—C6—N1—C29	52.5 (3)
C9—C10—C11—C12	0.0 (4)	C5—C6—N1—C29	177.9 (3)
C10—C11—C12—C7	0.7 (4)	C7—C6—N1—C2	178.0 (2)
C8—C7—C12—C11	−0.8 (4)	C5—C6—N1—C2	−56.6 (3)
C6—C7—C12—C11	178.7 (2)	C3—C4—N2—O1	−0.8 (3)
N1—C2—C13—C14	−57.7 (3)	C5—C4—N2—O1	−179.95 (19)
C3—C2—C13—C14	68.0 (3)	O2—C22—O1—N2	−3.3 (4)
N1—C2—C13—C18	122.3 (3)	O3—C22—O1—N2	178.33 (17)
C3—C2—C13—C18	−111.9 (3)	C4—N2—O1—C22	179.5 (2)
C18—C13—C14—C15	−0.6 (4)	O2—C22—O3—C23	−0.9 (4)
C2—C13—C14—C15	179.5 (3)	O1—C22—O3—C23	177.43 (19)
C13—C14—C15—C16	0.0 (5)	C24—C23—O3—C22	−133.2 (2)
C14—C15—C16—C17	0.0 (5)	C28—C23—O3—C22	51.7 (3)
C15—C16—C17—C18	0.6 (5)		
